# Network-Based Identification and Experimental Validation of Drug Candidates Toward SARS-CoV-2 *via* Targeting Virus–Host Interactome

**DOI:** 10.3389/fgene.2021.728960

**Published:** 2021-09-01

**Authors:** Jiansong Fang, Qihui Wu, Fei Ye, Chuipu Cai, Lvjie Xu, Yong Gu, Qi Wang, Ai-lin Liu, Wenjie Tan, Guan-hua Du

**Affiliations:** ^1^Institute of Materia Medica, Chinese Academy of Medical Sciences and Peking Union Medical College, Beijing, China; ^2^Science and Technology Innovation Center, Guangzhou University of Chinese Medicine, Guangzhou, China; ^3^Clinical Research Center, Hainan Provincial Hospital of Traditional Chinese Medicine, Guangzhou University of Chinese Medicine, Haikou, China; ^4^MHC Key Laboratory of Biosafety, National Institute for Viral Disease Control and Prevention, Beijing, China

**Keywords:** network-based identification, COVID-19, network proximity, natural product, protein-protein interaction

## Abstract

Despite that several therapeutic agents have exhibited promising prevention or treatment on Coronavirus disease-2019 (COVID-19), there is no specific drug discovered for this pandemic. Targeting virus–host interactome provides a more effective strategy for antivirus drug discovery compared with targeting virus proteins. In this study, we developed a network-based infrastructure to prioritize promising drug candidates from natural products and approved drugs *via* targeting host proteins of Severe Acute Respiratory Syndrome Coronavirus 2 (SARS-CoV-2). We firstly measured the network distances between drug targets and COVID-19 disease module utilizing the network proximity approach, and identified 229 approved drugs as well as 432 natural products had significant associations with SARS-CoV-2. After searching for previous literature evidence, we found that 60.7% (139/229) of approved drugs and 39.6% (171/432) of natural products were confirmed with antivirus or anti-inflammation. We further integrated our network-based predictions and validated anti-SARS-CoV-2 activities of some compounds. Four drug candidates, including hesperidin, isorhapontigenin, salmeterol, and gallocatechin-7-gallate, have exhibited activity on SARS-COV-2 virus-infected Vero cells. Finally, we showcased the mechanism of actions of isorhapontigenin and salmeterol *via* network analysis. Overall, this study offers forceful approaches for *in silico* identification of drug candidates on COVID-19, which may facilitate the discovery of antiviral drug therapies.

## Graphical Abstract

*In silico* identification of drug candidates on COVID-19 via targeting virus–host interactome.

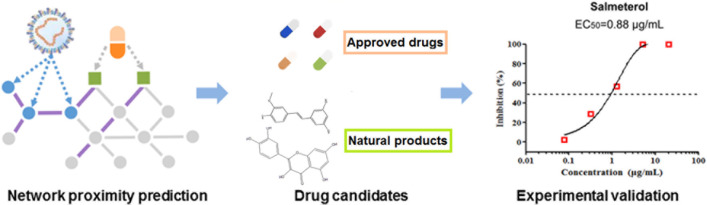

## Introduction

Coronavirus disease-2019 (COVID-19), which is caused by Syndrome Coronavirus 2 (SARS-CoV-2), is still posing an unprecedented threat to public health and development of economic society (Del Rio and Malani, 2020). As of March 3, 2021, the ongoing global outbreak of SARS-CoV-2 has resulted in 115.2 million infected cases and caused more than 2,500,000 deaths worldwide (Yang T. et al., 2020). Despite of increasing research and clinical trials against COVID-19, there is currently no definite effective agent for confirmed patients. Generally speaking, promising drug candidates for coronavirus can be classified as virally targeted agents (e.g., papain-like protease inhibitors; Wu C. et al., 2020) and host-targeted agents (e.g., angiotensin-converting enzyme 2 inhibitors; Li and De Clercq, 2020). However, the common resistance to virally targeted agents may become a major obstacle for developing effective therapies against SARS-CoV-2 (Mason et al., 2018). Additionally, mutation of the virus, virus-induced secondary infections, and adverse reactions from drugs also bring various uncertainties and create challenges to the efficacy of virally targeted agents (Chen et al., 2020). In contrast, host-directed therapy that may modulate the virus–host interactome potentially offers durable, broad-spectrum treatment modalities for viral infections (Zumla et al., 2016). For example, transmembrane serine protease 2 (TMPRSS2) inhibitors (e.g., camostat) were reported as potential treatments for COVID-19 since host cell factors such as TMPRSS2 and ACE2 are critical for successful virus replication during SARS-CoV-2 infection (Hoffmann et al., 2020a,b; Wang et al., 2021). Thus, it is of paramount importance to explore new drug therapy that targets SARS-CoV-2 host proteins.

Drug repurposing plays a key role in effective drug discovery strategy for the COVID-19 pandemic, which is able to significantly reduce the time and cut down the expense in comparison with developing drugs from scratch (Riva et al., 2020; Wu C. et al., 2020; Galindez et al., 2021). Excepting for drug repurposing from approved drugs that own preferable pharmacokinetics as well as pharmacodynamics profiles, natural products from traditional Chinese medicine have been demonstrated as alternative abundant sources for identifying potential treatment strategies (Fang et al., 2018). Indeed, several natural products have been clinically tested for COVID-19 and some of them have shown the therapeutic potential (Verma et al., 2020; Song et al., 2021). For instance, baicalin and pectolinarin have been reported to block the proteolytic activity of SARS-CoV-2 3CLpro (Jo et al., 2020). Taken together, identification of promising drug candidates from natural products and approved drugs could offer effective treatment and strategy against COVID-19.

Recent multi-omics technologies (e.g., proteomics) and network medicine approaches have advanced the understanding of the molecular virus–host–drug mechanisms and promoted the research and development of drug discovery (Bojkova et al., 2020; Gordon et al., 2020; Shen et al., 2020). For example, Bojkova et al. (2020) determined the proteomics profile of host cells infected with SARS-CoV-2, while Shen et al. (2020) presented proteomic and metabolomic profiling of patient sera in COVID-19. Moreover, multiple network-based methodologies (e.g., network proximity) help identify therapeutic agents *via* assembling drug–target interactions (DTIs) and disease proteins in the human protein–protein interactome (Cheng et al., 2019; Wu Q. et al., 2020; Zhou et al., 2020). Overall, a huge massive multi-omics data and latest progresses in network-based methodologies present unprecedented opportunities for accelerating target identification for drug discovery against COVID-19.

**FIGURE 1:**
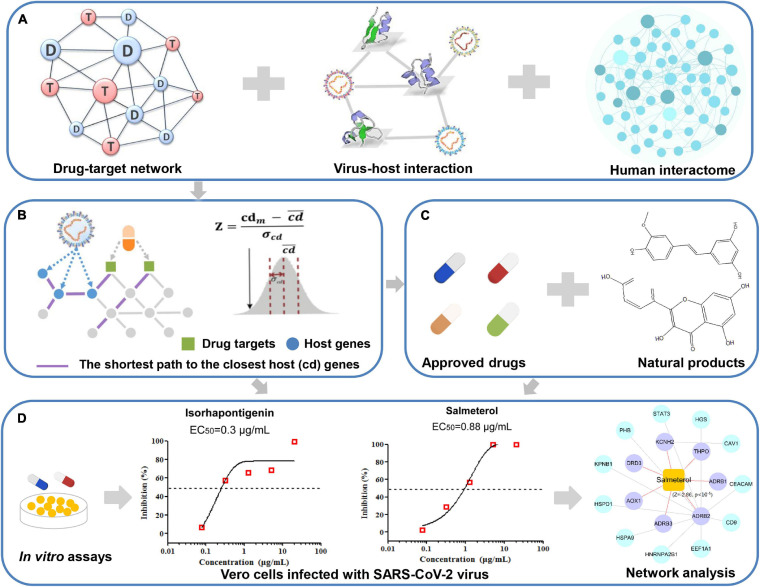
A schematic diagram illustrating network methodology and validation for *in silico* prediction of drug candidates against COVID-19. Integration of the drug–target network, virus–host interaction, and human interactome **(A)**. Network proximity approaches **(B)**. *In silico* prediction of drug candidates including approved drugs and natural products **(C)**. *In vitro* validation of potential candidates and network analysis inferring the mechanism of action **(D)**.

In this work, we constructed a network-based infrastructure to prioritize potential drug candidates toward the SARS-CoV-2 virus (Figure 1). Firstly, we manually collected three sets of high-quality host proteins from proteomics of SARS-CoV-2 and relevant experimental literature. To identify the potential drug candidates (approved drugs and natural products) against COVID-19, we built the associations between each compound and COVID-19 *via* measuring the interplay between the virus–host interaction network and drug targets within the human protein–protein interactome. Moreover, we integrated our network-based results and further validated several promising drug candidates. Finally, we selected typical drug candidates to infer their anti-SARS-CoV-2 mechanism of actions *via* network analysis. The whole framework of our study is seen in Figure 1.

## Materials and Methods

### Building the Human Protein–Protein Interactome

In this study, five types of comprehensive human protein–protein interactions (PPIs) including a yeast two-hybrid system, protein three-dimensional interactome, literature-derived kinase–substrate interactions, literature-derived signaling networks, and affinity-purification mass spectrometry (AP-MS) were integrated from 15 widely used databases. The elaborate descriptions of constructing the human interactome can be found in our recent studies (Cheng et al., 2019; Fang et al., 2020a).

### Construction of the Drug–Target Network for Approved Drugs

To build a comprehensive drug–target network for existing drugs, high-quality physical DTIs were integrated from six authoritative databases, including DrugBank database (v4.3; Wishart et al., 2018), the PharmGKB database (Barbarino et al., 2018), Therapeutic Target Database (TTD, v4.3.02; Yan et al., 2018), ChEMBL (v20; Gaulton et al., 2017), BindingDB (Gilson et al., 2016), and IUPHAR/BPS Guide to PHARMACOLOGY (Harding et al., 2018). Reported binding affinity data comprising dissociation constant (*K*_*d*_), inhibition constant/potency (*K*_*i*_), median effective concentration (*EC*_50_), or median inhibitory concentration (*IC*_50_) equal to or less than 10 μM were applied to define a physical DTI. Only those physical DTIs conforming to the following criteria were retained in our study: (i) protein targets owning a unique UniProt accession number; (ii) protein targets labeled as “reviewed” in the UniProt database; and (iii) protein targets marked as Homo sapiens species. Consequently, we assembled 15,367 DTIs interacting 1,608 approved drugs with 2,251 unique human targets/proteins.

### Construction of the Compound–Protein Network for Natural Products

To construct a compound–protein network for natural products, we further integrated DTIs from several kinds of data sources. First, we collected direct DTI databases derived from ChEMBL (v21; Gaulton et al., 2017) and BindingDB (accessed in September 2017; Gilson et al., 2016), as well as indirect DTI databases obtained from STITCH5 (Szklarczyk et al., 2016), the Herbal Ingredients Target Database (Ye et al., 2011), and the Traditional Chinese Medicine Integrated Database (Huang et al., 2018). Second, we also integrated the experimentally validated DTIs from an in-house database that was collected from over 2,000 natural product-specific pharmacological references (Huang et al., 2019). After filtering all the DTIs according to the criteria mentioned in our previous research (Fang et al., 2017c), 38,220 DTIs connecting 3,882 natural products and 5,643 protein targets were finally preserved.

### Network Proximity

Given *T*, the set of host protein targets related with disease, and *C*, the set of drug targets for a compound, *d*_TC_ (the nearest distance) means the average nearest route distance of all the proteins in the host protein set (*T*) to drug targets for a compound (*C*) within the human protein interactome, as below (Guney et al., 2016):

$$\left\langle d_{TC}\right\rangle =\frac{1}{||T||+||C||}\left(\sum_{t\in T}{\text{min}}_{c\in C}d\left(t,c\right)+\sum_{c\in C}{\text{min}}_{t\in T}d\left(t,c\right)\right)$$

where d(t,c) means the nearest route distance between host protein *t* and drug target c.

To evaluate the network length between *T* and *C*, we randomly extracted two sets of proteins from the human protein interactome that are same with the original protein sets in terms of size and degree distribution. After repeating this process for 1,000 times, the *Z*-score (*z_d*) was calculated as follows:

$$z_{d}=\frac{d-\bar{d}}{\sigma_{d}}$$

where *d* and $\bar{d}$ are *d*(*t*,c) and the mean, and σ_*d*_ represents the standard deviation of the reference distribution.

*p* values were computed during the permutation test. For a given host protein set, compound–disease (COVID-19) pairs were regarded as significantly proximal if they satisfied the condition (*Z* < −2 and *p* < 0.05). We further defined that a drug or natural product was a promising candidate against COVID-19 if it was predicted as positive by at least two host protein sets of three.

### Building the Virus–Host Interactome

The virus–host protein interaction networks of coronavirus (CoV) in our study were obtained from three latest public literature (Bojkova et al., 2020; Gordon et al., 2020; Zhou et al., 2020). Firstly, Zhou et al. integrated more than 100 high-quality host proteins which were validated with experiments for network-based drug reposition against SARS-CoV-2 (Zhou et al., 2020). Additionally, Gordon et al. (2020) systematically mapped the physical interactions between SARS-CoV-2 proteins and human protein targets utilizing AP-MS, and they identified 332 high-confidence SARS-CoV-2-human PPIs. Bojkova et al. (2020) revealed a proteomics profile of the cellular response to SARS-CoV-2 virus-infected human cells and identified 489 host proteins associated with SARS-CoV-2. Altogether, we assembled three host protein sets, resulting in 906 host proteins for further analysis (Supplementary Table 1).

### Cell Culture, Virus, and Compounds

Vero cells were purchased from the American Type Culture Collection (ATCC, CCL-81) and cultured in Dulbecco’s modified Eagle’s medium (DMEM, Gibco, Grand Island, NE, United States) supplemented with 10% fetal bovine serum (FBS, Gibco) in a humidified incubator of 5% CO_2_ atmosphere at 37°C. Subsequently, cells were digested with trypsin (at the concentration of 0.25%) and 1 × 10^4^ cells in 100 μl of complete medium were uniformly seeded in each well of 96-well plates. After incubating for 24 h, Vero cells were infected with virus or treated with drug. The infectious virus (C-Tan-nCoV Wuhan strain 01) utilized in this study was a SARS-CoV-2 virus strain, which was isolated from clinical cases. All drugs were provided by the Chinese Academy of Medical Sciences and Peking Union Medical College.

### Antiviral Activity Assay

*In vitro* antiviral efficiency of promising candidates against SARS-COV-2 virus on the Vero cell line was evaluated. Specifically, cells (at a density of 1 × 10^4^ cells/well) were plated into 96-well plates. After 24 h growth, Vero cells were pretreated with the drug candidates for 1 h. Subsequently, Vero cells were infected with the SARS-COV-2 virus (100 PFU/well) at a MOI of 0.01 for 2 h at 37°C. Following incubation, cells were maintained in medium with various concentrations of drugs for 48 h. Total viral RNA was quantified and analyzed utilizing the RT-PCR after extracting from the supernatant of the Vero cells.

## Results

### The Correlation Analysis of Multiple Host Gene Sets

**FIGURE 2:**
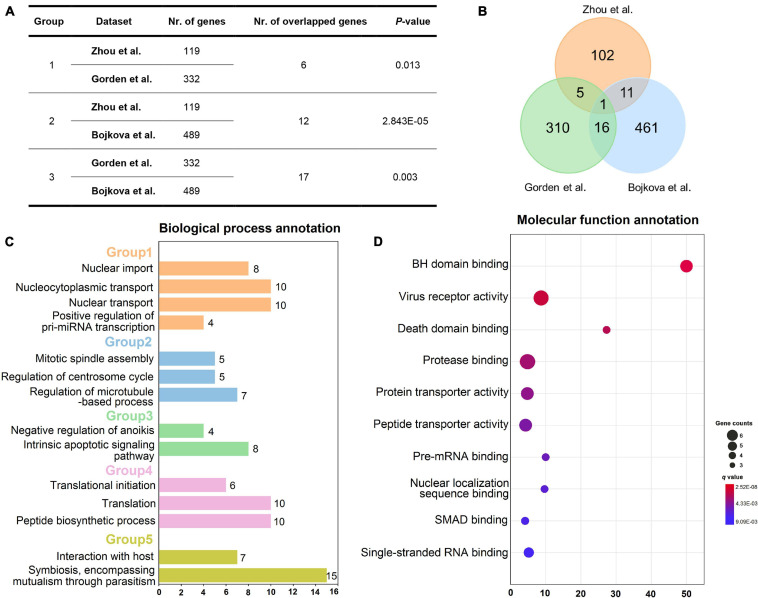
Overview of three host gene sets and enrichment analysis of gene set from Zhou et al. Correlation analysis of three different host gene sets **(A)**. Venn diagram showing the number of three host gene sets as well as their overlaps **(B)**. Biological process annotation results of the 119 host gene set of Zhou et al. **(C)**. Five biological process groups are displayed in various colors. Molecular function annotation results of the 119-host gene set from Zhou et al. **(D)**. Note: Nr. of genes: number of genes in each host gene set; Nr. of overlapped genes: number of overlapped genes between each two host gene sets. The annotation results **(C,D)** were obtained *via* using Cluego plugin in Cytoscape software (v3.2.1).

To decipher whether these gene sets share positive correlations at a biological level, the correlation analysis was performed on the three sets of host genes utilizing Fisher’s exact test approach. As presented in Figures 2A,B, the host genes from Zhou et al. are significantly correlated with those from Gorden et al. since they share six overlapped genes (*p* = 0.013, Fisher’s exact test). Moreover, it looks apparently that there are high correlations between gene sets from Gorden et al. and Bojkova et al. as they have the largest number of common genes, with the *p*-value of 0.003. The total number of their overlapped genes has reached up to 17, including *SRP54*, *GDF15*, *IMPDH2*, *DNMT1*, *TARS2*, *DCTPP1*, *RHOA*, *GGCX*, *DDX21*, *RAB5C*, *DDX10*, *PRIM2*, *CRTC3*, *PSMD8*, *ALG11*, *FBN1*, and *TOR1A*. Also, there are 12 overlapped host genes between gene sets from Zhou et al. and Bojkova et al. (*p* = 2.843E-05, Fisher’s test). Taken together, these host gene sets do own significant positive correlations, indicating the rationality of the selected gene datasets.

To further elucidate the specific mechanism of actions underlying these host genes, we performed biological process and molecular function annotations using 119 high-quality experimental validated host genes from Zhou et al. As exhibited in Figure 2C, these host genes are involved in five groups of biological processes, such as nuclear import and interaction with the host. Interestingly, a recent study has demonstrated that SARS-CoV-2 efficiently blocks STAT1 and STAT2 nuclear import in order to impair transcriptional induction of IFN-stimulated genes (ISGs; Miorin et al., 2020). Additionally, SARS-CoV-2 is well-known to interact with host cells and employ the host cell receptor (ACE2) for cell entry (Hoffmann et al., 2020b; Walls et al., 2020). Likewise, according to the enrichment analysis of molecular function (Figure 2D), these host genes may participate in the regulation of virus receptor activity and single-stranded RNA binding.

### Drug–Target Network Analysis for Natural Products and Approved Drugs

**FIGURE 3:**
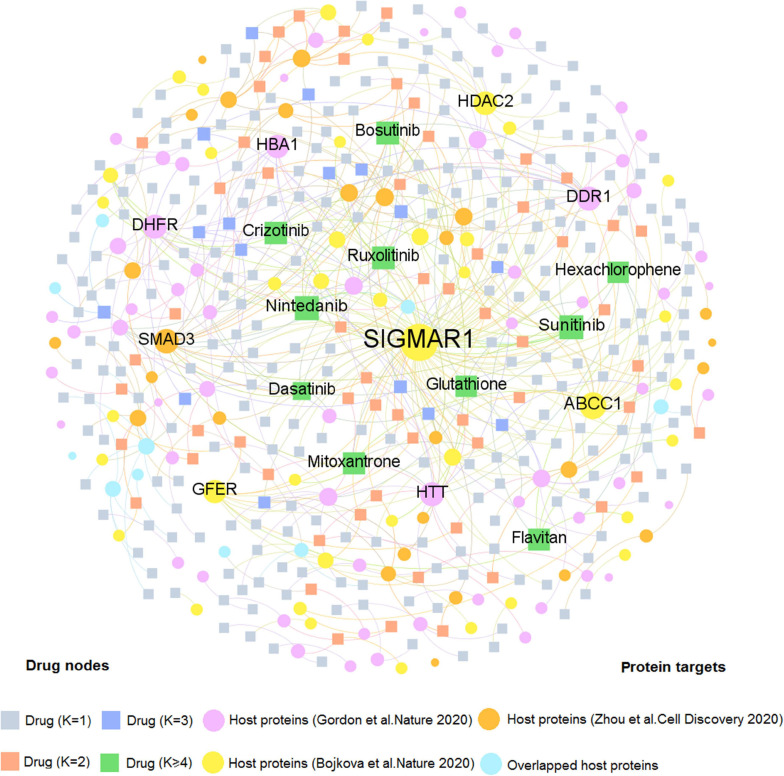
A bipartite drug–target network for approved drugs from the Drugbank database. This network contains 490 DTIs interacting 331 drugs with 137 human host proteins. The dot stands for the host protein target while the square node represents the drug. The label font size and node size are proportional to degree (connectivity). Only labels of the top 10 drugs and targets with the highest degree are displayed.

We firstly developed two comprehensive drug–target (D–T) networks through integrating natural products and approved drugs that specifically targeted coronavirus host proteins. As displayed in Figure 3, the D–T network focusing on approved drugs comprises 490 DTIs connecting 331 drugs to 137 human host proteins, with the average degree of 1.48 on each drug. The host protein with the largest degree is SIGMAR1 (*D* = 83), followed by ABCC1 (*D* = 21), and SMAD3 (*D* = 13). Emerging evidence indicates that these targets play vital roles in COVID-19. For example, SMAD3 is the main transcription factor for TGF-β1 signals and acting on TGF-β-mediated Smad2/3 signaling may prevent or treat pulmonary fibrosis in COVID-19-infected cases (Chen et al., 2020). Moreover, as one of the top 10 host proteins in the DTI network, HDAC2 is a potential regulator of ACE2 in the human lung that serves as an important part for SARS-CoV-2 virus to bind with and enter host cells (Pinto et al., 2020). Meanwhile, 10 approved drugs targeting host proteins have degree (*K*) higher than 5: nintedanib (*K* = 11), sunitinib (*K* = 11), ruxolitinib (*K* = 8), bosutinib (*K* = 8), crizotinib (*K* = 7), flavitan (*K* = 6), dasatinib (*K* = 6), mitoxantrone (*K* = 6), hexachlorophene (*K* = 5), and glutathione (*K* = 5). Notably, recent evidence has demonstrated high potential (e.g., ruxolitinib and mitoxantrone) with COVID-19 therapies (Goker Bagca and Biray Avci, 2020; Lokhande et al., 2020). For instance, ruxolitinib has been reported to exert potential effectiveness on COVID-19 *via* inhibiting the JAK/STAT pathway (Goker Bagca and Biray Avci, 2020).

**FIGURE 4:**
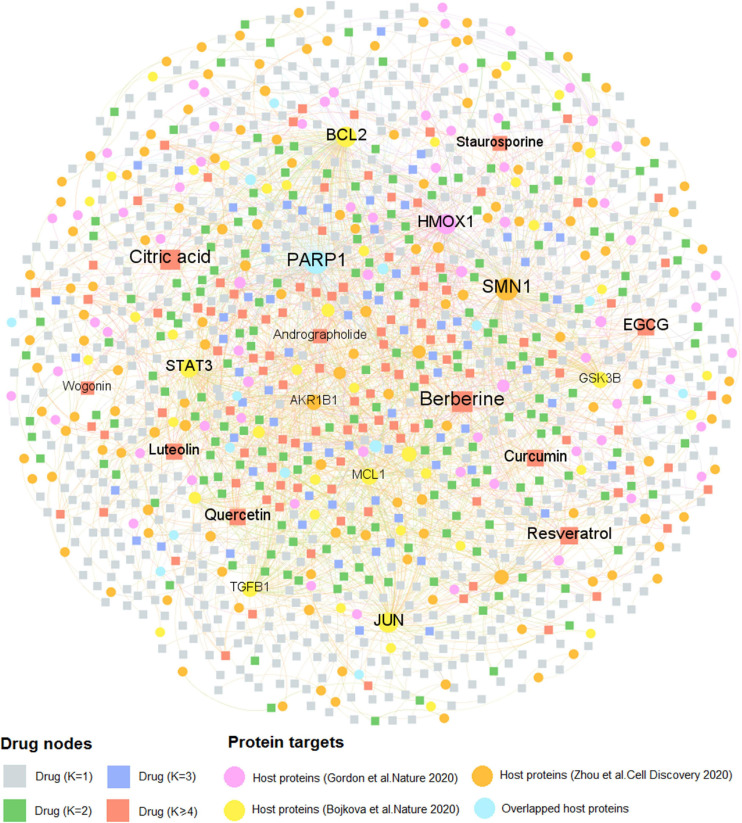
A bipartite compound–protein network for natural products. This network comprises 2,002 interactions covering 997 natural products with 259 human host proteins. The dot stands for the host protein target while the square node represents the natural product. The label font size and node size are proportional to degree (connectivity). Only labels of the top 10 natural products and proteins with the highest degree are displayed.

Similarly, Figure 4 comprises 2,002 interactions connecting 997 natural products with 259 human host proteins after removing ions and organic solvents. The average target degree (*K*) of a compound is 2.01, while the average compound degree (*D*) of a host protein is 7.73. Among the 997 natural products, the top 10 with the greatest target degree are berberine (*K* = 28), citric acid (*K* = 23), resveratrol (*K* = 23), epigallocatechin gallate (EGCG, *K* = 22), curcumin (*K* = 21), quercetin (*K* = 20), luteolin (*K* = 18), staurosporine (*K* = 13), andrographolide (*K* = 12), and wogonin (*K* = 11). Literature reveals that these natural products show the promising efficacy toward COVID-19. Taking resveratrol as an example, it inhibits the replication of SARS-CoV-2 virus in cultured Vero cells with an EC_50_ value of 4.48 μM (Yang M. et al., 2020). Thus, resveratrol could be considered as a potential therapeutic during the treatment against SARS-CoV-2. Besides, network analysis suggests that curcumin interacts with 21 host proteins and recent study demonstrates that curcumin may be assessed to inhibit cellular entry and replication of SARS-CoV-2 virus as well as to benefit for the damage of pneumocytes that are associated with COVID-19 (Soni et al., 2020), indicating its potential treatment option for patients with coronavirus disease.

Interestingly, we found that the average degree (*K* = 2.01) of natural products in the compound-CoV host protein network is slightly higher than that of approved drugs (*K* = 1.48) in the drug–target network (*P* = 1.97 × 10^–5^, one-sided Wilcoxon test), suggesting the “multi-target” effect of natural products. The detailed information of these two DTI networks is found in Supplementary Table 2. As the COVID-19 natural products and approved drugs could act on multiple host proteins according to the network analysis, thus, it is likely that most of the drug candidates may own the therapeutic potential toward COVID-19. Taken together, drug–target, or compound–protein network analyses contribute to the network-based identification of drug candidates and promote to further discover the potential therapeutic agents for COVID-19 through investigating both approved drugs and natural products.

### Network-Based Identification of Drug Candidates for COVID-19

The notion of the proposed network-based methodology posits that systematic identification and characterization of the virus–host interactome among the comprehensive human interactome network could facilitate effective therapeutic development for COVID-19. Here, we applied the advanced network proximity approach to measure the relevance between compounds and COVID-19 through assembling DTI networks, HCoV host proteins, and human PPI network. In total, 229 approved drugs were computationally identified to be associated with COVID-19 according to the criteria mentioned above. After searching for the previous literature, we found that 13 out of 229 approved drugs (Supplementary Table 3) were confirmed to possess anti-SARS-CoV-2 effects while 52 out of 229 approved drugs were reported to exert antiviral properties. Since cytokine storm is considered as one of the major pathological characteristics in patients infected with SARS-CoV-2 virus (Patra et al., 2020; Stebbing et al., 2020; Tian et al., 2020), we further sought evidence of anti-inflammation for these drug candidates and found that 113 out of 229 approved drugs were verified against inflammation. Taken together, there are 139 out of 229 (60.7%) approved drugs (Supplementary Table 3) that have been confirmed to possess anti-SARS-CoV-2, antiviral, or anti-inflammatory effects.

**FIGURE 5:**
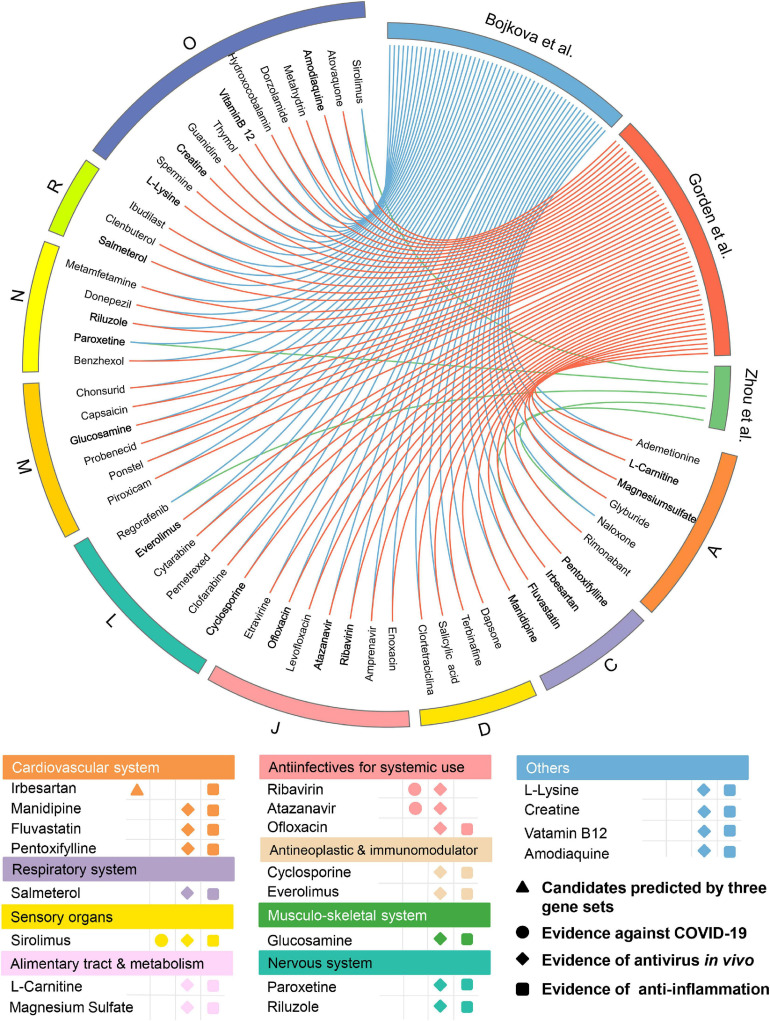
Network-based *in silico* prediction of approved drugs against COVID-19. Circos plot exhibiting 53 potential drug candidates identified by network proximity analysis. The full version exhibiting all the predicted approved drugs (*p* < 0.05) is provided in Supplementary Table 3. All drugs are grouped by their first-level Anatomical Therapeutic Chemical Classification (ATC) codes. Twenty best drug candidates were selected based on multiple studies from antivirus *in vivo* and anti-inflammation. Note: A: alimentary tract and metabolism; C: cardiovascular system; D: dermatologicals; J: anti-infectives for systemic use; L: antineoplastic and immunomodulating agents; M: musculo-skeletal system; N: nervous system; R: respiratory system; and O: others.

Among the 139 approved drugs, we selected 53 drug candidates, including 3 with experimental evidence of anti-SARS-CoV-2 effects and 50 that were satisfied with the following (Figure 5) factors: (i) confirmed antiviral effects; (ii) owning anti-inflammatory properties; and (iii) strength of the network-based prediction. As presented in Figure 5, 53 approved drugs can be grouped into 9 categories according to the first level of the Anatomical Therapeutic Chemical Classification (ATC) code. There are seven drugs for anti-infectives for systemic use, occupying the largest proportion (13.2%) among all the approved drug classes except “Others.” Interestingly, we found that several well-known anti-SARS-CoV-2 drugs (e.g., ribavirin and atazanavir) which belong to anti-infectives for systemic use were predicted to have significant associations on COVID-19 *via* our approach. For instance, atazanavir (*Z*-score = −2.815, by Bojkova et al. host protein set) was identified as a promising candidate, which has been reported to inhibit the replication of SARS-CoV-2 virus, alone or together with ritonavir in both Vero cells and a human pulmonary epithelial cell line (Fintelman-Rodrigues et al., 2020). We next systematically retrieved available evidence of antiviral effects *in vivo* for the 50 drug candidates to narrow up the scope of potential approved drugs (Figure 5). Among them, 19 network-predicted drugs were reported to exert antiviral effects *in vivo*. Additionally, potential candidates will also be kept if it was simultaneously predicted to be positive by all the three host gene sets. Altogether, 20 drug candidates were preserved for further validation (Figure 5).

**FIGURE 6:**
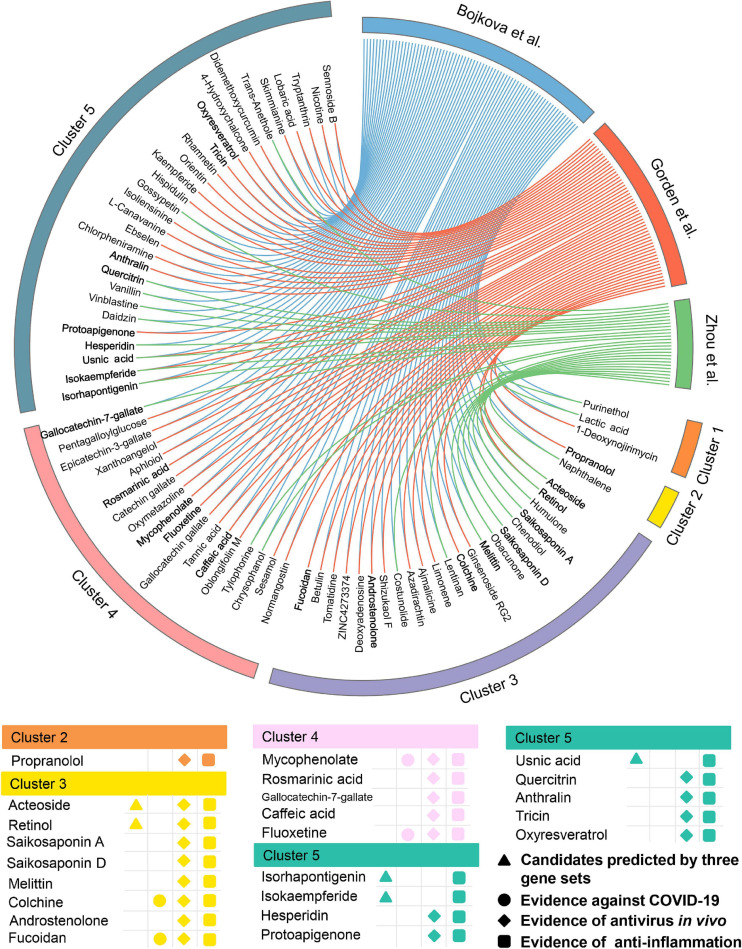
Network-based *in silico* prediction of natural products against COVID-19. Circos plot exhibiting 74 predicted natural products identified by network proximity analysis. The full version exhibiting all the predicted natural products (*p* < 0.05) is provided in Supplementary Table 4. All natural products are clustered by their scaffold structures. Twenty-three best natural product candidates were selected based on multiple studies from antivirus *in vivo* and anti-inflammation.

Meanwhile, 432 natural products were predicted to be associated with COVID-19.76 out of them (17.6%) were reported to show anti-antiviral effects, while 156 of them (36.1%) were confirmed to own anti-inflammatory properties (Supplementary Table 4), indicating a high hit rate of our network proximity approach. Likewise, 74 potential natural products were selected (Figure 6) according to the same subject matter expertise above. Scaffold analysis reveals that these natural products can be divided into five clusters. Among them, cluster 5 has the largest number of natural products (*n* = 29), followed by cluster 3 (*n* = 22), and cluster 4 (*n* = 18). Indeed, a recent study has demonstrated that fluoxetine (cluster 4, *Z*-score = −3.153, by Bojkova et al. host protein set), a well-known antidepressant, could efficiently inhibit the entry and propagation of the SARS-CoV-2 virus in both Calu-3 and Vero E6 cells (Schloer et al., 2020). We next turned to focus on 23 potential natural products (Figure 6) with both of public evidence, including antiviral *in vivo and* anti-inflammatory effects. Literature evidence shows that 4 out of 23 natural products are confirmed to exert anti-SARS-CoV-2 effects, including colchicine, fluoxetine, mycophenolic acid, and fucoidan. For example, mycophenolic acid, a highly effective immunosuppressant which owns broad antiviral activity against different viruses, has been reported to exhibit a high antivirus activity (EC_50_ = 0.87 μM) on SARS-CoV-2-infected VeroE6/TMPRSS2 monolayers (Kato et al., 2020). Similarly, fucoidan, commonly used in medicines and nutritional supplements for their healthy functions, also shows the inhibitory activity against SARS-CoV-2 virus with concentration of 15.6 μg/ml (Song et al., 2020). Detailed information of the predicted natural products is provided in Supplementary Table 4.

Taken together, our network-based approach offers novel and promising approved drugs and natural products with multiple supportive evidence for anti-SARS-CoV-2 effects, which deserves to be verified with various experimental assays or even randomized controlled clinical trials in the future.

### Evaluation of *in vitro* Efficacy of Drug Candidates on SARS-CoV-2

**FIGURE 7:**
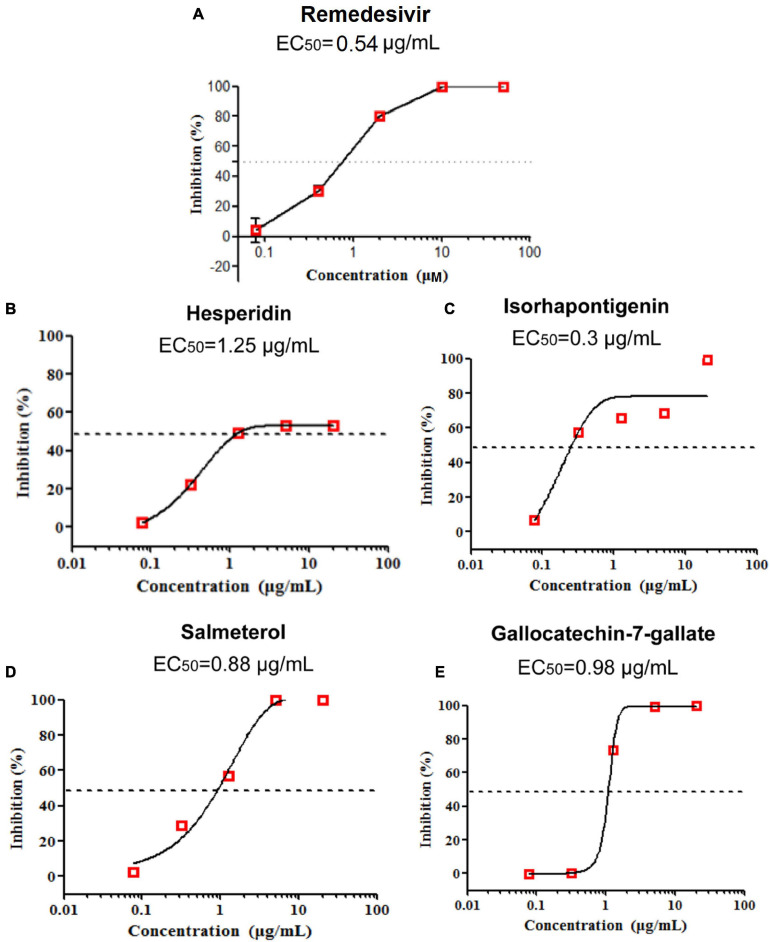
Remdesivir was used as the positive control drug **(A)**. The anti-SARS-CoV-2 activity of three natural products (hesperidin, isorhapontigenin, and gallocatechin-7-gallate) and one approved drug (salmeterol) *in vitro* **(B–E)**.

To test the anti-SARS-CoV-2 activity of potential drug candidates against COVID-19, we firstly excluded drugs and natural products with confirmed efficacy on COVID-19 and only selected those with specific anti-inflammatory and antiviral properties *in vivo* for further assay validations. After applying subject matter expertise criteria mentioned above, 19 natural products and 17 approved drugs (Figures 5, 6) were consequently obtained for testing anti-SARS-CoV-2 activity using RT-qPCR. During the process, remdesivir was used as the positive control drug (Figure 7A, EC_50_ = 0.54 μg/ml). In total, four promising candidates including hesperidin, isorhapontigenin, salmeterol, and gallocatechin-7-gallate were demonstrated to show inhibitory activity on SARS-CoV-2 virus-infected Vero cells. As presented in Figure 7, hesperidin (a flavanone glycoside isolated from various citrus fruits, such as orange; Küçükler et al., 2021) restrained the growth of the SARS-CoV-2 virus with an EC_50_ value of 1.25 μg/ml (Figure 7B) while isorhapontigenin (originated from plants and fruits, such as Chinese herb Gnetum cleistostachyum; Chu et al., 2020) inhibited the replication of virus with an EC_50_ value of 0.3 μg/ml (Figure 7C). As a citrus bioflavonoid, hesperidin possesses a variety of biological activities such as antioxidant, anti-inflammatory, and antivirus properties (Ding et al., 2018; Afzal et al., 2020). Recently, several computational methods have predicted hesperidin binds with the key proteins of the SARS-CoV-2 virus, including the “spike” protein and the main protease (Bellavite and Donzelli, 2020). In addition, salmeterol and gallocatechin-7-gallate both exhibited antiviral effects at EC_50_ values of 0.88 and 0.98 μg/ml, respectively, (Figures 7D,E). As a novel host cdc2-like kinase 1 inhibitor, gallocatechin-7-gallate which was isolated from *P*. clypearia (Li et al., 2016) exhibited potent *in vivo* and *in vitro* activities against influenza virus (Li et al., 2018). For instance, gallocatechin-7-gallate could significantly improve the survival rate of the mice that were infected with H1N1 virus and reduce the lung virus titer, indicating a promising drug candidate for antiviral therapy (Li et al., 2018). Taken together, the high activities of four promising drug candidates, especially isorhapontigenin in the antiviral experimental assays, imply that these compounds may act as promising drug candidates for SARS-CoV-2, which deserve to be further exploited in the future.

### Case Study: Mechanism of Action to Salmeterol and Isorhapontigenin on COVID-19

**FIGURE 8:**
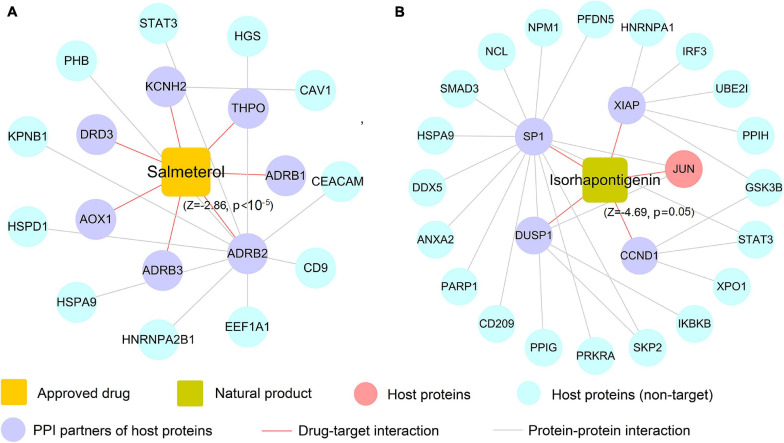
The predicted associations of approved drug (salmeterol) and natural product (isorhapontigenin) on COVID-19. A drug–target network for salmeterol connecting 11 corresponding targets of host proteins and 7 protein–protein interaction (PPI) neighbors **(A)**. A drug–target network for isorhapontigenin interacting with one host protein and 25 PPI neighbors **(B)**. Drug–target networks were constructed based on the host gene set of Zhou et al.

In this section, we selected salmeterol (an FDA-approved drug for asthma) and isorhapontigenin (a well-known natural product) to further decipher their mechanism of actions on COVID-19. Since COVID-19 is an infectious respiratory system disease with common clinical manifestations of fever and dry cough (Tay et al., 2020), respiratory system drugs such as salmeterol are naturally developed as the potential treatment options for infected patients. As depicted in Figure 8A, salmeterol targets on seven non-n-COV host proteins (ADRB1, ADRB2, ADRB3, AOX1, DRD3, KCNH2, and THPO), meaning that it may not act on COVID-19 *via* directly targeting COV host proteins. After assembling its PPIs, 11 PPI partners (e.g., CEACAM1 and STAT3) were added to this network and salmeterol was computationally predicted to show noticeable correlations on COVID-19 (*Z* = −2.86, *p* < 10^–5^). Interestingly, recent study has highlighted salmeterol as a potential drug against SARS-CoV-2 infection *via in silico* machine learning approach (Nunnari et al., 2020). However, the detailed molecular mechanism remains unclear. Figure 8A suggests that salmeterol could interact with STAT3 through integrating PPIs, providing the potential anti-SARS-CoV-2 mechanism of salmeterol. Indeed, STAT3 has been reported to affect ACE2 expression, which may be considered as a potential target for treating COVID-19 patients (Matsuyama et al., 2020; Shamir et al., 2020). Thus, it is likely that salmeterol could fight against COVID-19 *via* acting on STAT3.

Similarly, isorhapontigenin, a natural bioactive compound isolated from various plants and fruits (such as Chinese herb *Gnetum cleistostachyum* and grape), has shown several physiological activities including antiviral and anti-inflammatory effect *in vitro* and *in vivo* (Chu et al., 2020). The exact molecular mechanism against COVID-19 by isorhapontigenin is still indistinct. Network analysis (Figure 8B) suggests that isorhapontigenin interacts with one host protein and 20 PPI partners (e.g., CD209 and PARP1). Network proximity predicted isorhapontigenin with high association with COVID-19 (*Z* = −4.69, *p* = 0.005). CD209 has been reported to bind with S-RBD and also mediate SARS-CoV-2 S-pseudotyped virus entry, which can be regarded as alternative receptors for the SARS-CoV-2 virus (Amraie et al., 2020). Additionally, increasing evidence shows that the activation of PARP1 is the terminal point among a sequence of events culminating in patient mortality which should serve as the focus of COVID-19 immunotherapy (Badawy, 2020). Figure 8A shows that isorhapontigenin targets CD209 and PARP1 through protein–protein interaction, implying an anti-SARS-CoV2 mechanism of isorhapontigenin on COVID-19. Notably, we found that isorhapontigenin could also target STAT3, suggesting an alternative mechanism of isorhapontigenin on COVID-19. Taken together, aforementioned examples indicate that systems pharmacology-based network analyses offer new perspective for elucidating the anti-SARS-CoV-2 mechanisms of drug candidates. However, experimental validation is warranted to confirm its underlying mechanisms in future.

## Discussion

As COVID-19 is still raging all over the world, it has made a serious threat to the global life health. Nevertheless, currently no effective medicine has been found for this pandemic. In this tough campaign to SARS-CoV-2, it is urgently needed to identify the potential prevention or therapeutic medications. In this work, we proposed a novel network-based infrastructure to prioritize promising approved drugs or natural products for COVID-19: (i) developing two global D–T networks for both natural products as well as approved drugs, respectively, (ii) establishing potential associations of natural products and approved drugs on COVID-19 *via* the network proximity approach by assembling compound–protein network, and virus–host interactome within the human protein interactome, (iii) validating the anti-SARS-CoV-2 activity of the promising drug candidates on SARS-COV-2 virus-infected Vero cells, and (iv) deciphering the underlying molecular mechanisms of drug candidates on COVID-19 with network analysis. Taken together, we demonstrated that the network-based framework can offer forceful tools for uncovering the potential therapeutic strategies for COVID-19 through exploiting the great wealth of natural products as well as existing drugs and help us better understand their mechanisms of actions on COVID-19.

In comparison with the previous studies (Fang et al., 2017a,b; Zhou et al., 2020), several remarkable improvements can be highlighted. First, integrating high-quality host proteins from proteomics of SARS-CoV-2 virus within the human protein interactome complemented the current known disease proteins of COVID-19 and improved the predictive accuracy on potential drug candidates. In this manner, we can therefore establish potential associations on COVID-19 for those drug candidates that interacted with only one or none of host proteins (e.g., salmeterol). Moreover, our network-based infrastructure comprehensively predicted potential natural products for the treatment of COVID-19, as previous study only focused on repurposing approved drugs (Zhou et al., 2020). Finally, we confirmed the anti-SARS-CoV-2 effects for several drug candidates (e.g., salmeterol and isorhapontigenin) on SARS-COV-2 virus-infected Vero cells.

However, several limitations of this work should be recognized. First, despite that a large number of DTIs from publicly available databases and reported literature were integrated for both approved drugs and natural products, the incompleteness of current D–T networks may still be inevitable. Recent literature has demonstrated that the importing of the computational predicted DTIs inferred by balanced substructure–D–T network-based inference may benefit for the incompleteness of the known drug–target networks (Wu et al., 2016, 2017). In addition, drug targets that represent nodes among cellular networks are often intrinsically coupled with both therapeutic and adverse effects since drugs could activate or inhibit the functions of proteins (including agonists vs. antagonists; Bedi et al., 2016). Thus, the present network proximity approach in our study cannot distinguish therapeutic (antiviral) effects from side effects in the absence of specific pharmacological actions of drug targets as well as unclear functional outcomes of virus–host interactions. Integrating functional genomic assays or extensive expression profiles of disease genes (e.g., downregulating or upregulating) will notably enhance the accuracy of the network-based methodologies further (Cheng et al., 2016, 2018). Finally, a gap may exist between the anti-SARS-CoV-2 effects at the cellular level and the true effect in COVID-19-infected patients. The identified drug candidates should be further validated in randomized controlled clinical trials or the large-scale patient longitudinal database (Fang et al., 2020a,b).

## Conclusion

The network-based infrastructure proposed in this study suggests efficient strategies for *in silico* identification of potential approved drugs and natural products against COVID-19, which can shorten the time and improve efficiency to prioritize potential drugs. If broadly applied, this network-based approach presented here can be also utilized for developing new drug candidates of other diseases as well.

## Data Availability Statement

The datasets presented in this study can be found in online repositories. The names of the repository/repositories and accession number(s) can be found in the article/Supplementary Material.

## Author Contributions

JF, A-LL, and G-HD conceived and designed the research. WT and FY carried out the experiments. QWu, CC, and LX performed the data analysis. YG and QWa revised the manuscript. JF and QWu wrote the manuscript. All the authors have read and approved the final manuscript.

## Conflict of Interest

The authors declare that the research was conducted in the absence of any commercial or financial relationships that could be construed as a potential conflict of interest.

## Publisher’s Note

All claims expressed in this article are solely those of the authors and do not necessarily represent those of their affiliated organizations, or those of the publisher, the editors and the reviewers. Any product that may be evaluated in this article, or claim that may be made by its manufacturer, is not guaranteed or endorsed by the publisher.

## Supplementary Material

Supplementary Table 1 Detailed information of three sets of host proteins obtained from latest public literature.

Supplementary Table 2 Detailed information of drug-target interaction network of approved drugs and compound-protein interaction network of natural products.

Supplementary Table 3 Detailed information of the network-based prediction results on 229 approved drugs and 20 drug candidates.

Supplementary Table 4 Detailed information of the network-based prediction results on 432 natural products and 23 drug candidates.
