# Supplementary material for: Network-Based Identification and Experimental Validation of Drug Candidates Toward SARS-CoV-2 via Targeting Virus–Host Interactome
Source: Front Genet. 2021 Sep 1;12:728960. doi: 10.3389/fgene.2021.728960 (PMC8440948; doi:10.3389/fgene.2021.728960)
Supplement: Supplementary Table 1 — Detailed information of three sets of host proteins obtained from latest public literature. [file Data_Sheet_1.zip › Supporting information/Supporting information.docx]

**Supplementary Data**

**Table S1.** Detailed information of three sets of host proteins obtained from latest public literature.

**Table S2.** Detailed information of drug-target interaction network of approved drugs and compound-protein interaction network of natural products.

**Table S3.** Detailed information of the network-based prediction results on 229 approved drugs and 20 drug candidates.

**Table S4.** Detailed information of the network-based prediction results on 432 natural products and 23 drug candidates.
